# Multiple Independent Origins of Tn*916*-Mediated Tetracycline Resistance in *Clostridium tetani* from a Confined Geographic Area

**DOI:** 10.3390/antibiotics15080745

**Published:** 2026-07-31

**Authors:** Chie Shitada, Motohide Takahashi, Makoto Kuroda

**Affiliations:** 1 Toxin and Biologicals Research Laboratory, Kumamoto Health Science University, 325 Izumi-machi, Kita-ku, Kumamoto 861-5598, Japan; shitada@kumamoto-hsu.ac.jp (C.S.); takahashi@kumamoto-hsu.ac.jp (M.T.); 2 The Chemo-Sero-Therapeutic Research Institute (KAKETSUKEN), 8-7 Shinshigai, Chuou-ku, Kumamoto 860-0803, Japan; 3 Department of Medical Laboratory Science, Faculty of Health Sciences, Kumamoto Health Science University, 325 Izumi-machi, Kita-ku, Kumamoto 861-5598, Japan

**Keywords:** *Clostridium tetani*, *tet*(M), tetracycline, Tn*916*, transposon, molecular phylogenetics, agricultural environment

## Abstract

**Background/Objectives:** Tetracycline resistance in Gram-positive bacteria has increased globally through horizontal gene transfer. Antimicrobial resistance genes in *Clostridium tetani*, the causative agent of tetanus, are rarely reported. Previously, we identified tetracycline resistance gene *tet*(M)-positive *C. tetani* strains in Japan, but their evolutionary origin and acquisition mechanism remain unclear. This study aimed to elucidate the evolutionary origin of *tet*(M)-positive *C. tetani* and clarify the mechanism of horizontal resistance gene acquisition. **Methods:** Complete genome sequences of six *tet*(M)-positive *C. tetani* strains were determined using hybrid assembly of short-read (Illumina) and long-read (Oxford Nanopore) sequencing. Core genome single-nucleotide variant (SNV) analysis was performed to determine phylogenetic relationships. Tn*916* element analysis of the *tet*(M) gene identified the origin of resistance genes and potential donor bacteria. **Results:** Based on core genome SNV analysis, all six *tet*(M)-positive strains belonged to Clade 1-2. The Tn*916* element was approximately 18 kb and highly conserved; however, insertion sites differed significantly among strains. Phylogenetic analysis of the *tet*(M) gene revealed at least three distinct variants with different origins. **Conclusions:** Multiple independent acquisitions of Tn*916*-mediated *tet*(M), rather than clonal propagation, drove resistance emergence in geographically confined *C. tetani* populations. These findings support the notion that tetracycline resistance emerged through multiple independent horizontal transfer events involving Tn*916*. This pattern suggests that persistent environmental selective pressure, rather than clonal expansion, has shaped resistance dissemination, highlighting the importance of genomic surveillance and systematic monitoring to track the emergence of antimicrobial resistance in environmental and clinical settings.

## 1. Introduction

Tetanus is an infectious disease caused by the tetanus neurotoxin produced by *Clostridium tetani* [1,2]. *C. tetani* is an obligate anaerobic, Gram-positive rod bacterium that is widely distributed in soil [1,3]. Infection is established through tissue damage sites such as wounds, puncture wounds, and burns [1,2]. This bacterium exists as spores in the environment and exhibits high levels of resistance to heat, desiccation, and disinfectants [1,3]. Due to the high environmental resistance of these spores, *C. tetani* survives in the environment for extended periods and is maintained as an infection source for humans and animals [1,2,3]. In the treatment of tetanus, antimicrobial agents are used in addition to wound debridement, antitoxin therapy, and supportive care to suppress bacterial growth and prevent additional toxin production [2,4]. According to current guidelines, metronidazole is recommended as the first-line agent, while penicillin G is used as an alternative [2,4,5,6]. Additionally, although tetracycline-class antimicrobials are not first-line agents, they may be used as alternatives in specific situations (such as severe allergic reactions to penicillin and metronidazole) [1,5].

In recent years, an increase in tetracycline resistance in Gram-positive bacteria has been reported [7,8,9]. Particularly in *Streptococcus pneumoniae* and *Clostridioides difficile*, tetracycline-resistant strains have become a clinical problem and frequently carry the *tet*(M) gene [8,10,11]. Although tetracycline is not routinely used to treat these pathogens clinically, the widespread distribution of *tet*(M) suggests it functions as a reservoir for resistance gene dissemination through horizontal gene transfer (HGT) among diverse bacterial species. Tetracycline resistance genes are often located on mobile genetic elements such as plasmids, transposons, and insertion sequences, and are known to disseminate among bacterial species through HGT [7,10].

Tetracycline resistance includes multiple mechanisms such as drug efflux pumps, ribosomal protection proteins (RPPs), and drug-inactivating enzymes [12,13]. RPPs exhibit GTPase activity and confer resistance by binding to the ribosome and dissociating tetracycline [14,15,16,17]. Among RPPs, Tet(M) is the most representative and has been detected in a diverse range of Gram-positive bacteria, including *Streptococcus*, *Enterococcus*, and *Clostridium* species [12,14,15,18]. The *tet*(M) gene is widely disseminated in both Gram-positive and Gram-negative bacteria through mechanisms such as conjugative transposons (e.g., Tn*916*), transformation, and transduction, and plays an important role in global resistance dissemination [14,19].

We have previously reported five *tet*(M)-positive *C. tetani* strains isolated in Japan and determined the complete genome sequence of strain NIID-071400-001, revealing that an approximately 18 kb Tn*916* element containing *tet*(M) is inserted into the tetanus toxin plasmid pNIID-071400-001 [20]. Most of these strains were isolated from environmental soil in the southern region of Kumamoto Prefecture in Japan, and an association with the environment, such as nearby livestock farms, has been suggested.

In livestock farms, tetracycline-class antimicrobials are widely used for disease prevention and treatment, and these drugs are released into the environment through animal excreta [21,22]. Although the addition of tetracycline to livestock compound feed was banned in Japan in December 2019, since the primary habitat of *C. tetani* is soil, it is conceivable that past selective pressure in the agricultural environment promoted the acquisition of tetracycline resistance in *C. tetani* in the environment [20,23]. In subsequent investigations, we isolated another *tet*(M)-positive strain, KHSU-CS4-02, in this study. As a result, in addition to the five strains mentioned above, a total of six *tet*(M)-positive *C. tetani* strains have now been identified in Japan. It remains unclear whether these strains are the result of clonal spreading in a limited area within the prefecture, or the result of genetically distinct *C. tetani* independently acquiring *tet*(M). To date, there have been no reports that comprehensively compare the phylogenetic relationships and structure of *tet*(M)-containing mobile elements or their insertion sites at the whole-genome level among multiple *tet*(M)-positive *C. tetani* strains. Therefore, the evolutionary origin of *tet*(M)-positive *C. tetani* and the process of resistance gene acquisition remains insufficiently elucidated.

In this study, we determined the complete genome sequences and performed genomic analysis of the six *tet*(M)-positive *C. tetani* strains identified in Japan. We conducted structural comparisons of Tn*916* that contain *tet*(M), insertion site analysis, and molecular phylogenetic analysis of the *tet*(M) sequence, with the aim of elucidating the establishment process of *tet*(M)-positive *C. tetani* and the mechanism of resistance gene acquisition.

## 2. Results

### 2.1. Complete Genome Sequence Information on tet(M)-Positive C. tetani in This Study

**Figure 1:**
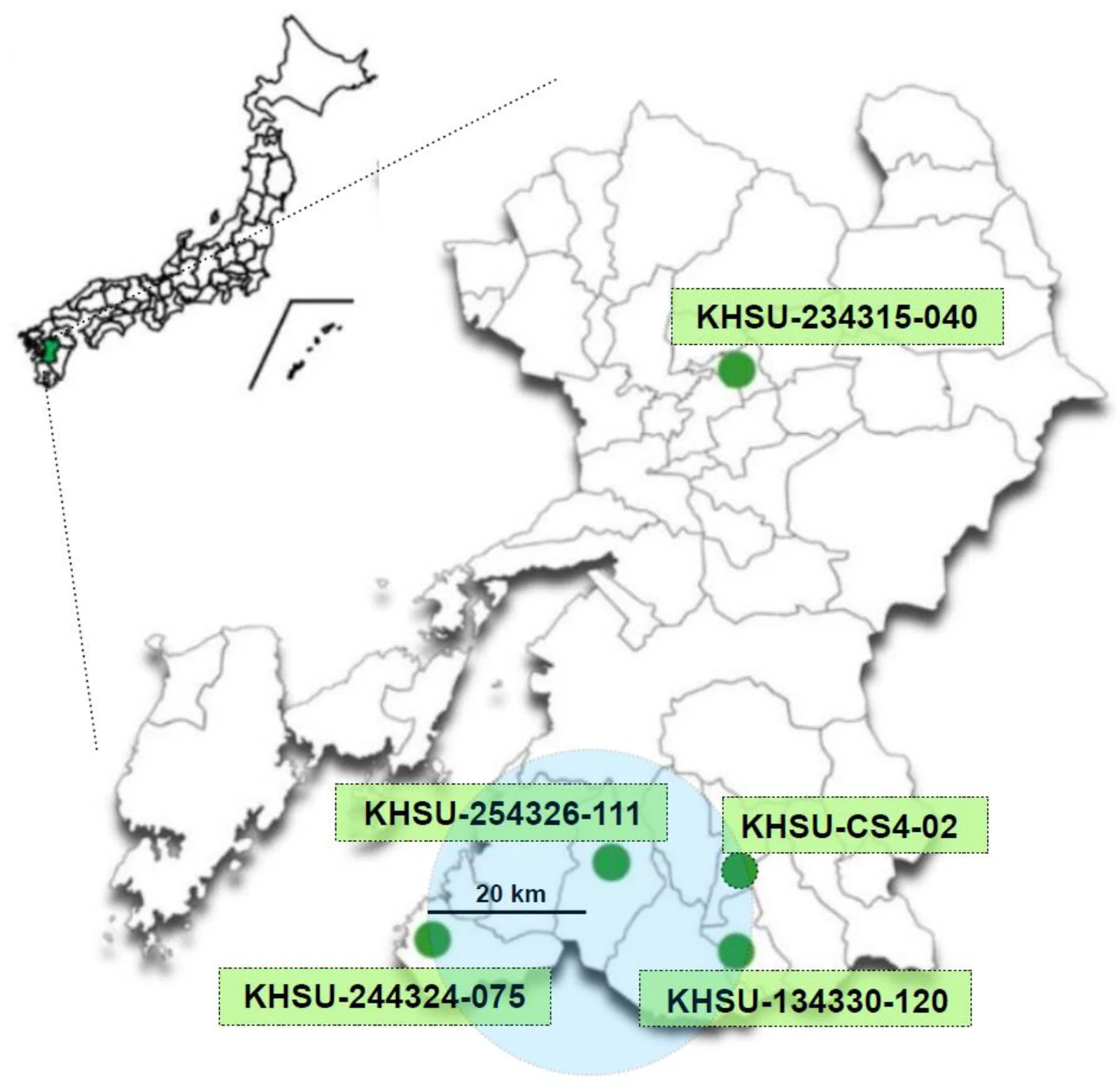
Isolate location of tetracycline-resistant *C. tetani* strains (green circles). Of the five strains, four were isolated within a 20 km radius in south of Kumamoto prefecture. The NIID-071400-001 strain was clinically isolated in another part of the prefecture in 2013 (Table 1).

We previously reported five *tet*(M)-positive *C. tetani* strains isolated domestically, and determined the complete genome sequence of strain NIID-071400-001, revealing that an approximately 18 kb Tn*916* element containing *tet*(M) is inserted into the tetanus toxin plasmid pNIID-071400-001 [20]. In addition to those five strains isolated in 2021, we conducted soil sampling at seven sites in 2025. *C. tetani* was isolated at four of the seven sites (57.1%), yielding five isolates in total. Among these five isolates, one strain (20.0%) was positive for *tet*(M) and was designated KHSU-CS4-02 (Table 1, Figure 1). Combined with the previously reported strains, a total of six *tet*(M)-positive *C. tetani* strains have now been identified in Japan. We performed hybrid assembly using short- and long-read sequencing to obtain complete genome sequences in this study, and the metadata and genomic information on these six strains is presented (Table 1).

### 2.2. Core Genome Single-Nucleotide Variant (SNV) Analysis

**Figure 2:**
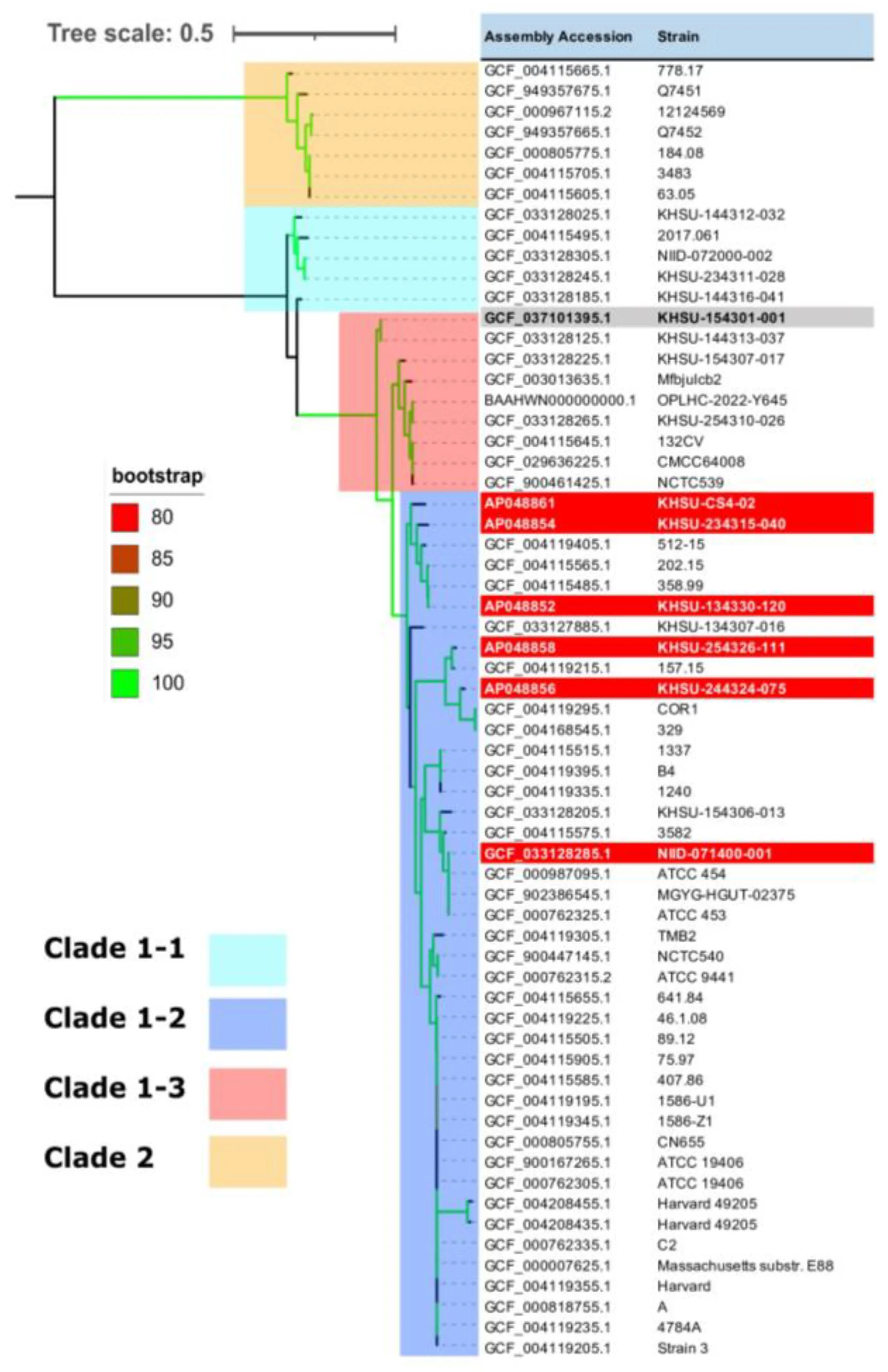
Core genome SNVs phylogenetic analysis of *C. tetani* strains including the six *tet*(M)-positive strains (highlighted in red).

For core genome SNV analysis, 1,934,282 bp corresponding to 69.1% of the entire genome was used, and 86,786 SNVs were detected. Phylogenetic tree analysis revealed that all six *tet(M)*-positive strains belonged to Clade 1-2 (Figure 2).

### 2.3. tet(M) Element Structure Analysis

**Figure 3:**
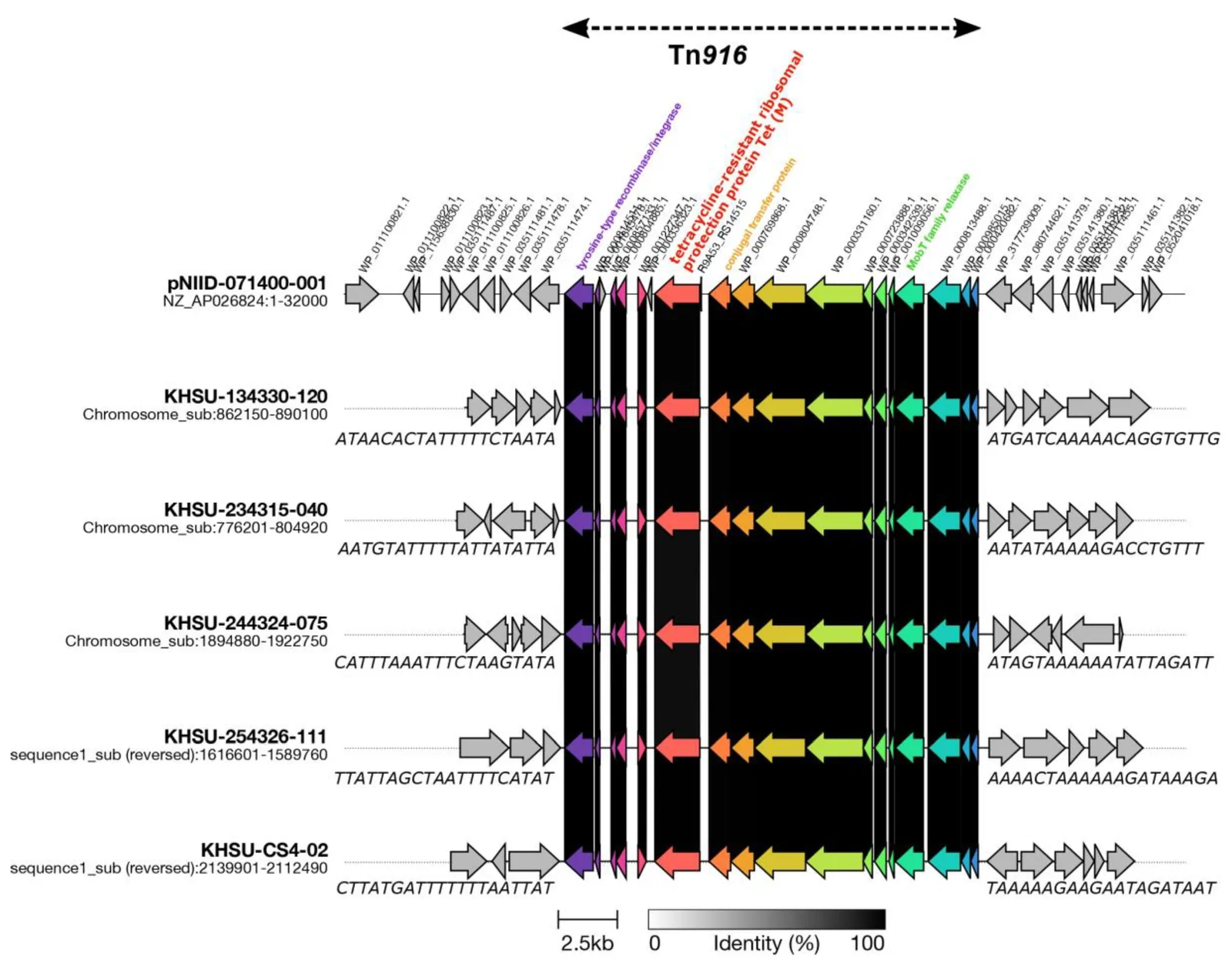
Comparative analysis of the horizontally acquired Tn*916* element (18 kb) including *tet*(M) among the six strains. Coding sequences showing above 95% homology are connected with the black shaded boxes between the genome alignments generated using the Clinker tool. Coding sequences represented by gray arrow boxes flank the region at the Tn*916* integration site in their chromosome DNA. No similarity was found at the flanking regions among the six strains, because their integration sites are completely different (see Figure 4). The nucleotide sequences of the Tn*916* insertion boundaries are shown outside the Tn*916* ORF diagram.

Comparative alignment of the regions flanking *tet*(M) in all six strains were highly conserved, and it was revealed that the mobile genetic element is equivalent to the approximately 18 kb Tn*916* element that contains the tyrosine-type recombinase-related genes previously reported in strain NIID-071400-001 (Figure 3).

### 2.4. Insertion Site Analysis of tet(M) Element

**Figure 4:**
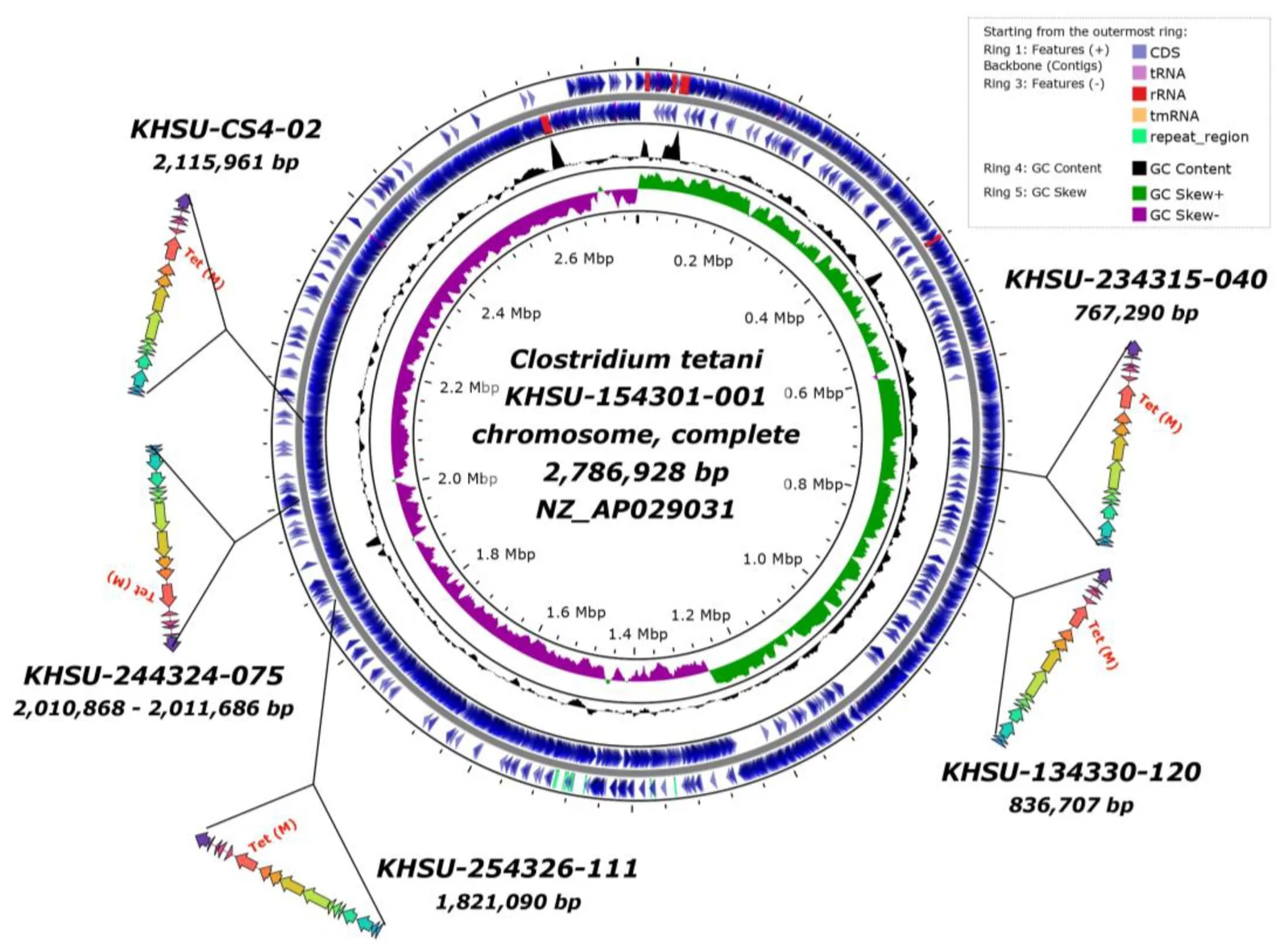
The Tn*916* element (18 kb) was found to be integrated into chromosomes at different sites among the five strains. The complete genome sequence of *C. tetani* KHSU-154301-001 (GenBank ID: NZ_AP029031) was used as a *tet*(M)-negative (Tn*916*-negative) reference. Possible integration sites of the Tn*916* element for respective strains are shown beyond the circular genome representation and include the direction of the Tn*916* element (see also Figure 3) and the integration position. The Tn*916* element in NIID-071400-001 was found to be integrated into the tetanus toxin plasmid pNIID-071400-001 (NZ_AP026824.1), which was previously reported [20], and is therefore not shown in this figure.

Comparative analysis of the homology in the flanking regions of the Tn*916* sequence in each strain revealed no homology at all (Figure 3). Therefore, we evaluated the Tn*916* insertion sites mapping onto the complete genome sequence of KHSU-154301-001 isolated at the Kumamoto Health Science University campus (GenBank ID: NZ_AP029031) as a reference strain. The results show that Tn*916* insertion sites differed among the strains. In NIID-071400-001, the site was located on the tetanus toxin plasmid, as previously reported, but in the five strains for which we determined the complete genome sequences in this study, it was inserted at different locations on the chromosome (Figure 4). Since Tn*916* insertion sites differed in each strain, the possibility of clonal expansion is likely to be low, suggesting that tetracycline resistance was independently acquired in each strain through Tn*916* via different routes and in different environmental contexts (Figure 4).

### 2.5. tet(M) Sequence Comparison and Phylogenetic Analysis

**Figure 5:**
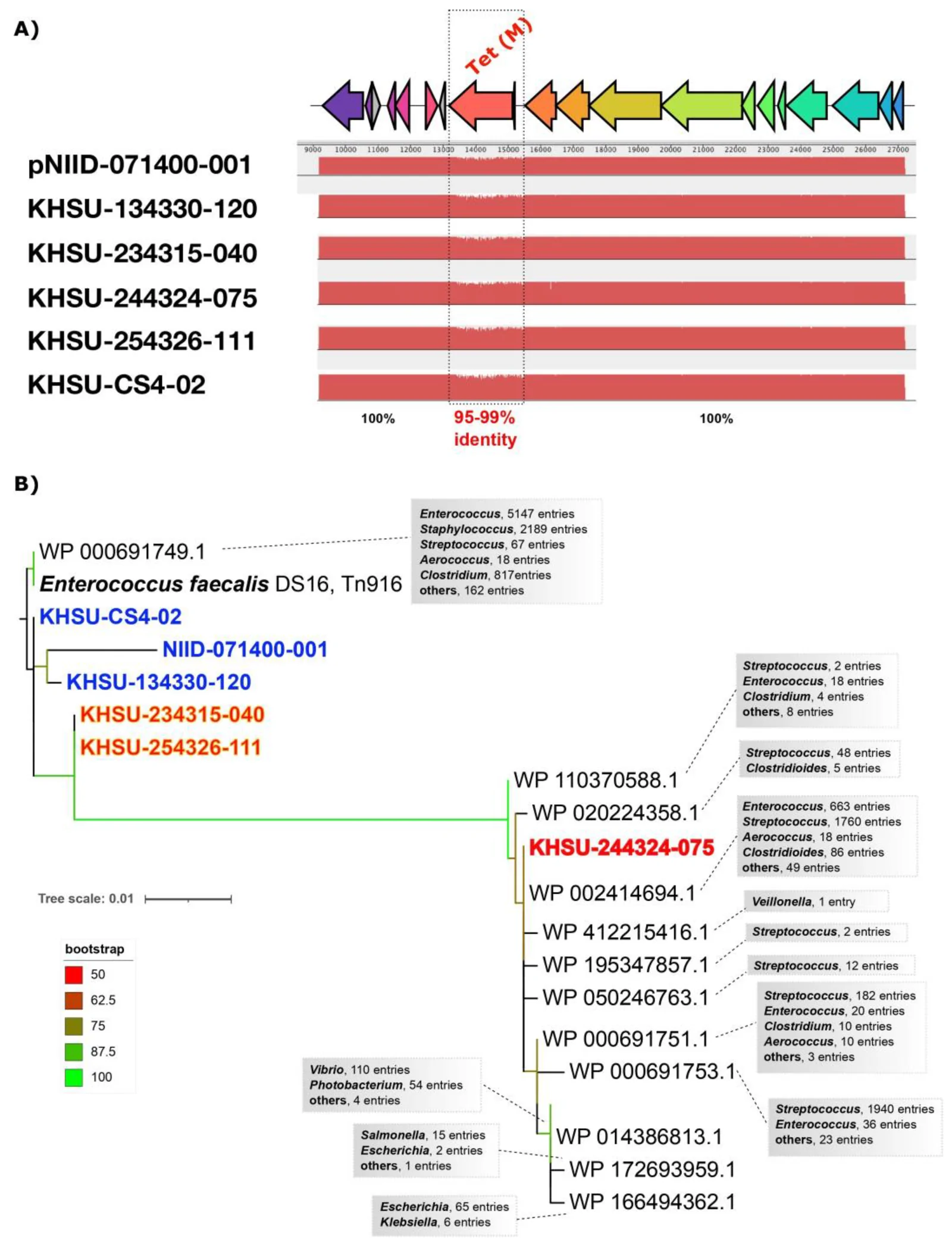
Genetic analysis of the *tet*(M) genes. (**A**) Pairwise nucleotide alignment of the Tn*916* element (18 kb, see also Figure 3) among the six strains suggested that the *tet*(M) gene exhibited a significantly reduced nucleotide identity (95–99%) compared with flanking regions on the element (100%). (**B**) Phylogenetic tree of Tet(M) amino acid homologs with 1000-fold bootstrap replicates (bootstrap values are shown at each branch). The tree demonstrates that most of the five *C. tetani* strains were classified in close association with Tn*916* in *E. faecalis* DS16 (the reference strain for Tn*916*) and WP_000691749.1, showing the largest entries for *Enterococcus*. It is suggested that these Tn*916* elements were acquired from *Enterococcus* species, subsequently, the *tet*(M) genes were adapted to the codon usage in *Clostridium* species. Secondly, KHSU-244324-075 has acquired the most prevalent *tet*(M) gene, similar to WP_002414694.1 and WP_166494362.1, showing links with the *Streptococcus* and *Escherichia* species, respectively.

The overall Tn*916* sequence showed a high degree of nucleotide sequence conservation. In contrast, the *tet(M)* gene region showed 95–99% homology, and some diversity was observed compared to the flanking regions (Figure 5A).

Phylogenetic analysis of Tet(M) amino acid sequences revealed that KHSU-CS4-02, which is highly similar to the Tn*916* element from the *E. faecalis* DS16 strain, is thought to be an original form acquired immediately after transmission from *Enterococcus*. In KHSU-254326-111/KHSU-234315-040, the *tet*(M) gene is hypothesized to be a type that has begun to adapt to the codon usage of the *C. tetani* genome (Figure 5B).

The Tet(M) of KHSU-244324-075 belonged to a widely disseminated type and a major cluster that includes Tet(M) from Gram-negative bacteria such as *Vibrio*, *Salmonella*, and *Escherichia*, in addition to those from *Streptococcus*/*Enterococcus* species (Figure 5B). Thus, the phylogenetic relationship of the *tet*(M) gene is thought to represent the evolution of three *tet*(M) variants with different temporal scales and origins.

## 3. Discussion

To date, reports of antimicrobial resistance genes in *C. tetani* are extremely limited [20,24], and the acquisition process remains largely unexplained. In our previous study, we reported that an approximately 18 kb Tn*916* element containing *tet*(M) is inserted into the toxin plasmid of strain NIID-071400-001 [20]. However, in this study, while similar Tn*916* elements were present in multiple strains, the insertion sites differed among all strains (Figure 4). This suggests that these elements may function as mobile genetic factors within the *C. tetani* population.

The most important finding in this study is that *tet*(M)-positive *C. tetani* strains isolated in Kumamoto Prefecture could not be explained as a single resistant clone, even when the previously reported strain NIID-071400-001 was included. In general, regional dissemination of resistant bacteria is often explained by the propagation of a single clone [25]. However, in this study, the core genome SNV analysis revealed that *tet*(M)-positive strains did not form a single cluster (Figure 2), and furthermore, the insertion sites of the Tn*916* elements containing *tet*(M) differed among the strains (Figure 4). These results support the possibility that the *tet*(M)-positive *C. tetani* identified domestically are not a single resistant clone with a common ancestor, but have emerged through independent evolutionary pathways.

In the phylogenetic analysis, the Tet(M) derived from KHSU-244324-075 belonged to a major cluster that includes Tet(M) from Gram-negative bacteria species (Figure 5B). In contrast, the remaining five strains formed a cluster closely related to Tet(M) from *C. tetani* and other *Clostridium* species. Tet(M) is known to move among diverse bacterial species through conjugative transposons and integrative and conjugative elements (ICEs) [26,27], and the results of this study suggest that *tet*(M) acquisition in *C. tetani* occurred through multiple donor sources, rather than a single HGT event [28,29]. Furthermore, *tet*(M) has been detected in nearly one-fifth of *C. difficile* clinical isolates globally [11], suggesting that *tet*(M) is a globally disseminated resistance determinant whose environmental and clinical reservoirs warrant continued surveillance.

Most of the strains analyzed in this study were isolated from soil within a 20 km radius in the southern region of Kumamoto Prefecture, an area with active livestock farming (Figure 1). Tetracycline-class antimicrobials were widely used in Japan as feed additives and growth promoters prior to the regulatory ban in December 2019 [30,31]. Although the extent of their use in this specific region of Kumamoto Prefecture is unknown, tetracycline remains a major antimicrobial agent in Japanese livestock agriculture. While tetracycline feed additives were banned in December 2019, tetracycline is still widely used as a veterinary pharmaceutical for therapeutic purposes, accounting for approximately 45% of total veterinary antimicrobial sales and primarily used in swine (70%) and cattle [32]. Antimicrobial residues from livestock can be released into the environment through animal excreta [21,33]. Tetracycline resistance genes, including those on mobile genetic elements such as Tn*916*, have previously been detected in soils from livestock-intensive regions [34,35]. This persistent environmental contamination may have created sustained selective pressure favoring independent acquisitions of *tet*(M)-carrying Tn*916* elements in *C. tetani* strains inhabiting farm soil, potentially explaining the multiple acquisition events observed in geographically confined populations.

Farm environments are known to function as reservoirs of resistance genes [22,36,37]. Since the animal species raised, rearing conditions, and antimicrobial use history differ among individual farms, each farm likely maintains a relatively independent microbial ecosystem [38]. This spatial compartmentalization could explain why *tet*(M)-positive strains, despite originating from a geographically limited area, display independent evolutionary origins and show variation in their genomic structures rather than representing a single clonal expansion.

While the approximately 18 kb Tn*916* element containing *tet*(M) showed high conservation, the *tet*(M) gene itself exhibited relatively large nucleotide sequence diversity (Figure 5A). This suggests that while the entire mobile genetic element is conserved to function as a mobile factor (i.e., is under weak selective pressure against mutations), the *tet*(M) gene itself permits sequence variation. This is a phenomenon that has already been reported and, in the reviews by Clewell and colleagues and related papers, the entire Tn*916* sequence shows relatively low concordance rates (~94–95%) for *tet*(M) sequences among different bacterial species and between different transposons (Tn*916* vs. Tn*1545*), whereas the core regions such as recombinase (int-Tn) are nearly identical (≥99%) (19). This is emphasized as important evidence for “diversification through horizontal dissemination of *tet*(M)” [19,27]. Tet(M) is a protein with GTPase activity that binds to the ribosome and has a structure and function similar to elongation factor (EF-G). It is thought that *tet(M)* undergoes mutations to better fit the structure of the host bacterial ribosome, thereby affording it tetracycline resistance [39,40,41,42].

This study is limited to analysis of six strains, and we were unable to directly identify the donor bacteria of *tet*(M). Additionally, we did not experimentally verify the actual mechanisms of HGT. Nevertheless, this study is the first report to comprehensively compare the phylogenetic relationships, mobile genetic element structure, and insertion sites at the whole-genome level for multiple *tet*(M)-positive *C. tetani* strains.

The above indicates that the *tet*(M)-positive *C. tetani* identified in Japan were not established through propagation of a single resistant clone, but rather that it is highly likely that strains with different genetic backgrounds independently acquired *tet*(M). Furthermore, the phylogenetic analysis results for *tet*(M) suggest the existence of resistance gene acquisition routes through multiple donor sources. Because this genomic analysis relies on environmental isolates derived from soil, we could not specify the exact environment where selective pressure occurred—specifically, whether resistance arose in the soil itself or within the intestinal tract of livestock. Further investigation is required to identify the specific conditions driving this selection.

## 4. Materials and Methods

### 4.1. Bacterial Strains and Isolation

This study examined six *tet*(M)-positive *C. tetani* strains; five previously identified strains and one newly isolated strain KHSU-CS4-02. The previously identified strains include NIID-071400-001, which was clinically isolated in Kanagawa Prefecture in 2013, and four environmental soil isolates (KHSU-134330-120, KHSU-234315-040, KHSU-244324-075, and KHSU-254326-111) obtained from Kumamoto Prefecture in 2021. The newly isolated strain KHSU-CS4-02 was collected from soil near Hitoyoshi in the southern region of Kumamoto Prefecture in November 2025, an area of active livestock farming that is geographically close to the sampling site of strain KHSU-134330-120.

*C. tetani* isolation and identification was performed as previously described [20]. Briefly, soil samples of 1 g were collected from seven locations around Hitoyoshi at the surface layer and inoculated into heat-treated degassed cooked meat medium (CMM). After heating at 80 °C for 10 min to select for spores, cultures were incubated anaerobically at 37 °C for 24–72 h. Bacterial cells from the swarming zones were inoculated into CMM for pure culture isolation. *C. tetani* identification was confirmed by Gram staining using a RAPID ID 32 A (bioMérieux, Lyon, France). Tetracycline susceptibility was assessed by E-test (bioMérieux, Tokyo, Japan) following the methodology outlined by the Clinical and Laboratory Standards Institute (CLSI) for anaerobic bacteria [43]. Minimum inhibitory concentration (MIC) values were determined and reported as CLSI interpretive breakpoints are not currently established for *C. tetani* [43,44].

### 4.2. Whole-Genome Sequencing and Phylogenetic Analysis

Whole genome sequencing was performed using previously described methods [20]. The isolated strain was cultured in brain heart infusion (BHI) medium at 37 °C for 24 h, then centrifuged at 8000 rpm for 20 min, and the recovered cells were inactivated by phenol/chloroform treatment. The bacterial suspension was disrupted using ZR BashingBead Lysis Tubes (Zymo Research, Irvine, CA, USA), and DNA was purified using MinElute PCR Purification Kit (QIAGEN, Hilden, Germany). DNA sequencing libraries were prepared using QIAseq FX (QIAGEN) and sequenced using the iSeq 100 sequencer (Illumina, San Diego, CA, USA) with 150 bp paired-end reads. Additionally, a sequencing library was prepared using the Ligation Sequencing gDNA-Native Barcoding Kit 24 V14 (Oxford Nanopore Technologies, Oxford, UK). Sequencing was performed on a MinION Mk1b using an R10.4.1 flow cell controlled by MinKNOW v24.06.16, with base calling carried out using Dorado v7.4.14 [45]. Hybrid assembly of the Illumina and Nanopore reads using Unicycler v0.4.8 [46] yielded a circular complete genome sequence of a chromosome and plasmids. 

Genome annotation and taxonomic verification, including average nucleotide identity (ANI) calculations, were performed using DFAST v1.6.0 (https://dfast.ddbj.nig.ac.jp/, accessed on 1 July 2026) [47] and ResFinder 4.7.2 (https://genepi.food.dtu.dk/resfinder, accessed on 1 July 2026) [48]. The circular map of genome annotation was visualized using the Proksee website (https://proksee.ca/, accessed on 1 July 2026) [49]. Furthermore, core genome phylogenetic trees and pairwise single-nucleotide variants (SNVs) were determined using Parsnp v1.7.4 [50]. Phylogenetic relationships were evaluated using iQtree version 2.2.5 [51] with 1000-fold bootstrap replicates using the following parameters (iqtree2-s parsnp.snps.mblocks-alrt 1000-B 1000-nt 6). The phylogenetic tree was visualized using the iTOL website (https://itol.embl.de/, accessed on 1 July 2026) [52]. Pairwise alignment was performed with Clinker [53] and Mauve [54] to compare gene cluster similarity and nucleotide identity. SVG file formats were modified by Inkscape 1.1.1 for the figure illustrations.

## 5. Conclusions

This study reveals that *tet*(M)-positive *Clostridium tetani* in Kumamoto Prefecture did not emerge from a single resistant clone (Figure 2). Genomic analysis, varied Tn*916* insertion sites (Figure 4), and Tet(M) sequence variations (Figure 5B) suggest diverse genetic backgrounds and independent horizontal gene transfer events from multiple donor sources. We hypothesize that persistent environmental selective pressure from veterinary tetracycline use in this livestock farming-intensive region facilitated these recurrent acquisitions. Given that *C. tetani* resides primarily in the environment—with human or animal infections being incidental and transient—these findings underscore farm soils as dynamic reservoirs where diverse *C. tetani* populations independently acquire and evolve resistance via mobile genetic elements. Further large-scale environmental and epidemiological surveys in other areas are needed to elucidate the influence of livestock production and antibiotic use on the independent emergence of *tet*(M).

## Figures and Tables

**Table 1:** Tetracycline-resistant ribosomal protection protein Tet(M)-positive *Clostridium tetani* strains in this study.

Strain Name	Collection Date (YYYY-MM-DD)	Location	Isolation Source/Host	MIC to Tetracycline (µg/mL)	Genome Assembly Status	GenBank (Chromosome, Plasmid)	Coverage (>Q15)	N50, Nanopore Read Length (bp)	Reference
NIID-071400-001	2013-xx-xx	Japan: Kanagawa	Homo sapiens	64	Complete	NZ_AP026823.1, NZ_AP026824.1	—	—	[20]
KHSU-134330-120 *	2021-11-22	Japan: Kumamoto	soil	>256	Complete	AP048852, AP048853	×812	7696	this study
KHSU-234315-040 *	2021-01-25	Japan: Kumamoto	soil	12	Complete	AP048854, AP048855	×133	14,872	this study
KHSU-244324-075 *	2021-11-02	Japan: Kumamoto	soil	1	Complete	AP048856, AP048857	×94	10,819	this study
KHSU-254326-111 *	2021-10-02	Japan: Kumamoto	soil	128	Complete	AP048858, AP048859, AP048860	×305	12,225	this study
KHSU-CS4-02 *	2025-11-15	Japan: Kumamoto	soil	4	Complete	AP048861, AP048862	×207	14,513	this study

* BioProject: PRJDB42350.

## Data Availability

The complete genome sequence data of the five strains in this study have been deposited in the DNA Data Bank of Japan (DDBJ) and are publicly available, as shown in Table 1.
